# Mapping the landscape of professional learning communities for digitalization and STEM: a scoping review of evidence on composition and efficacy

**DOI:** 10.3389/fpsyg.2025.1696783

**Published:** 2025-12-04

**Authors:** Stefanie Hartmann, Robin Bardakcioglu, Steffen Schaal

**Affiliations:** 1 Institute for Biology, University of Education Ludwigsburg, Ludwigsburg, Germany; 2 Allvest GmbH, A Subsidiary of Allianz SE, Munich, Germany

**Keywords:** STEM-professional learning community, scoping review, digitalization, teachers’ collaboration, teaching development

## Abstract

**Introduction:**

The integration of advanced digital applications within the educational sector remains a significant challenge. It has been determined that extracurricular educational settings possess a considerable capacity to enhance and differentiate digital instruction. However, educators frequently require assistance to transition from the use of existing digital resources to the creation of their own, personalized, customized learning materials. Professional learning communities (PLCs) have been identified as a promising framework in which teachers can collaborate to develop innovative and practical concepts. While the potential of PLCs is widely recognized, research on this topic is fragmented. A multitude of definitions and operationalizations have been proposed regarding the structure and functioning of PLCs. This complicates a systematic investigation of their effectiveness and makes it difficult to distinguish empirically based findings from purely anecdotal reports. Consequently, a definitive evidence base for the design of effective PLCs remains elusive.

**Methods:**

The present study posits that the afore mentioned gap is addressed by means of a scoping review. Utilizing the PRISMA-ScR framework, a systematic analysis of German and English-language literature is conducted with the objective of mapping and synthesizing research on the composition, collaboration, and effectiveness of PLCs in the STEM field or in relation to digitalization.

**Results:**

The results of the study provide a structured overview of the key characteristics, structure, and success factors of PLCs. The present article elucidates the findings that emerged from a comparative and harmonising analysis of diverging PLCs.

**Discussion:**

The ensuing discussion pertains to the configuration, efficacious design, and execution of professional learning communities, in conjunction with their operational methodologies and investigative endeavours. This synthesis provides a foundation for the conceptualisation of future cooperative initiatives aimed at the development of STEM- or digital educational resources.

## 1 Introduction

The integration of digital technologies into STEM subjects (science, technology, engineering and mathematics) is progressing slowly in Germany. Based on the latest international Computer and Information Literacy Study (ICILS) conducted by Eickelmann et al. (2024), the pedagogical integration of digital technologies has shown substantial progress in Germany over the past five years. Despite this notable advancement, German students’ digital literacy decreased, and their performance remains in the middle ranks when compared internationally. This finding suggests that while significant strides have been made, Germany still faces ongoing challenges in fully leveraging and embedding digital tools into its educational practices, particularly when benchmarked against leading countries in this domain.

Apart from a few pilot projects, the use of digital applications lags far behind what is possible. However, non-school learning environments offer the opportunity to use digital tools to individualize and deepen learning processes (Schaal, 2020). Within the domain of STEM, the integration of digital technologies poses a multifaceted challenge (OECD, 2020), yet it also offers significant potential. In contradistinction to other domains, this field is distinguished by both its technological dynamism and the specific nature of the subject matter. The integration of simulations, measurement data acquisition systems, and programming environments, which is of didactic significance, necessitates a high level of subject-specific didactic and technical expertise (Nerdel and von Kotzebue, 2020; Mishra and Koehler, 2006) - a level that individual teachers are unlikely to possess in sufficient depth to ensure on their own. Furthermore, contemporary STEM teaching is increasingly focusing on research-based and discovery-based learning processes as well as design-based approaches (Thibaut et al., 2018).

According to a needs analysis in the *lernen:digital* project, teachers show a general willingness to use digital technologies, but they underestimate their own development competencies in this area (Hartmann et al., 2025). This creates a need for professional and collaborative support formats. The joint development, testing, and reflection of such complex, project-based learning scenarios within the framework of professional learning communities therefore appears to be a particularly promising approach to overcoming the specific challenges of digital change in STEM subjects. Increasingly being established as a central instrument of school and teaching development (Kansteiner et al., 2020; Bonsen and Rolff, 2006) professional learning communities represent a promising strategy.

### 1.1 Rationale for a scoping review

Professional learning communities (PLC) are groups of teachers who work together – often in collaboration with scientists, academics and other specialists – to continuously improve teaching (Kansteiner et al., 2020). They are characterized by long-term collaboration, a shared goal orientation and an intensive reflective dialogue (Bonsen and Rolff, 2006; Rolff, 2007; DuFour, 2004). The aim is not simply to exchange materials, but to co-construct new teaching concepts, particularly regarding the use of digital media (Little, 1990; Gräsel et al., 2006). Personal skills such as the ability to reflect, openness to new teaching concepts, and a willingness to cooperate are essential for successful participation in such a community (Kullmann, 2010). The groups are characterized by their flexibility in composition, with the potential for organization across schools and subjects. The role of the school administration in providing support is identified as a critical factor in the success of these groups (Rolff, 2014). The long-term structure of such groups has been shown to promote collective and individual learning in equal measure (Stoll et al., 2006).

DuFour and Eaker (1998) identify five characteristics they deem essential for professional learning communities. These include shared mission, (i) vision and values; (ii) collective inquiry; (iii) collaborative teams; (iv) action orientation and experimentation; and, finally, (v) continuous improvement. As this scoping study clearly shows, many PLCs base their work on these characteristics. Despite their high educational relevance, empirical research into professional learning communities is fraught with methodological difficulties. The use of terminology is inconsistent, theoretical approaches diverge, and distinctions from other forms of collaboration are unclear, which makes it difficult to draw reliable conclusions about their effectiveness (Bloh and Bloh, 2016; Warwas and Schadt, 2020). Current studies frequently encounter difficulties in clearly differentiating between results that are empirically proven and those that are based on experience (Lomos et al., 2011; Timperley et al., 2007; Stoll et al., 2006). Vescio et al. (2008) investigated the influence of PLCs on teaching practice and student performance. However, their analysis of 11 cases revealed a low level of empirical evidence, and they noted that the impact on student performance was hardly measured objectively. In addition, they recommended that research should seek to establish a clear definition, empirical validation, and better methods. However, to effectively implement this, a closer examination of the current landscape of PLCs is first required, moving beyond the scope of existing meta-analyses. As Kansteiner et al. (2020) observes, research on PLCs – including extant meta-analyses – frequently highlights an absence of clarity with regard to their empirical foundation and conceptual diversity. This situation calls for a systematic synthesis of the literature A scoping review is the most appropriate methodology for this purpose. In contradistinction to a systematic review, the purpose of which is to provide answers to narrow clinical questions, a scoping review is designed to map the extant literature in a broad field, identify key concepts, and uncover research gaps (Tricco et al., 2018). In light of the inconsistent terminology and divergent theoretical approaches observed in the field of PLC research (Bloh and Bloh, 2016), this methodological approach will facilitate the comprehensive mapping of PLC structures, collaborations, and research designs, unencumbered by the limitations imposed by a single, narrow research question.

### 1.2 Objectives

Based on the background outlined above, this study employs a scoping review. The goal is to examine the conceptualization and design of PLCs and to determine their effectiveness in order to subsequently design our own PLCs that deal with the development of digital tools for out-of-school learning environments, understood as learning activities intended by the school but carried out beyond school premises (Karpa et al., 2015). Given the conceptual diversity and lack of consistent empirical findings in PLC research, a Scoping Review is suitable to map the existing knowledge base, identify key concepts, and uncover gaps in the literature. The review method is characterized by a systematic approach and a broad search strategy that includes various types of contributions in order to obtain an overview of a broad field of research and identify gaps in knowledge (Tricco et al., 2018). This analysis aims to develop a nuanced understanding of the potential and challenges of PLCs in the context of digital transformation and in STEM (Carmi et al., 2022; Stegmann et al., 2022a, 2022b; Hartmann et al., 2025).

The purpose of this scoping review is to detail the structures, compositions, forms of cooperation, and aspects of (self-)efficacy of PLCs. The review will provide insights into how these communities are described in the literature, specifically in the context of teaching development in the STEM field or the development of digital products. In order to give a suitable question for scoping, the SPICE scheme was applied, a process developed by Booth (2006) to arrive at a precise research question. The present study is founded upon the PICO scheme developed by Lockwood et al. (2015), which was adapted for social and educational research. The SPICE scheme is predicated on the setting, which in this article is the school. The standpoint adopted herein is that of pedagogues within a professional learning community. Nevertheless, the emphasis on professional learning communities has undergone a substantial shift over the past three decades. This has resulted in an exponentially high number of PLCs that are eligible for investigation by means of scoping. The present study thus focuses on teachers who wish to enhance student learning in STEM subjects, including digitalization. The intervention under scrutiny is the professional learning community itself. The dimension of comparison cannot yet be incorporated into the formulation of the research question. The absence of a systematization of different PLCs thus far precludes the possibility of comparison between them. The evaluation of the SPICE scheme is focused on the enhancement of teaching through cooperative endeavors and collaborative construction. The subsequent research question was formulated for the scoping phase:

*What do existing studies reveal about the composition, collaboration, and research of professional learning communities (PLCs) that focus on teaching development in the STEM field or the creation of digital products?*

Sub-questions:

*What theory of professional learning communities do PLCs work with?*

*What organizational and personnel structures are described for PLCs?*

*What forms of collaboration are used?*

*What research approaches and designs are used to study PLCs?*

## 2 Methods

To answer the research questions, a scoping review was carried out. The scoping review process was conducted according to the PRISMA-ScR framework by Tricco et al. (2018) and recorded according to the specified guidelines. During the preparatory research, three scoping reviews or protocols were identified that deal with aspects of the present research topic (e.g., communities of practice in higher education (Gómez and Suárez, 2021), online communities for teachers’ shared understanding (Dille and Røkenes, 2021), or collaborative work in clinical settings (Shen et al., 2017)). However, none of these reviews explicitly addresses the topic of professional learning communities (PLCs) in its entirety. This preliminary work was used for conceptual delimitation and methodological orientation. The following section therefore describes the methodological process of literature selection and the approach taken to synthesis.

### 2.1 Eligibility criteria

As recommended by Tricco et al. (2018), the inclusion and exclusion criteria for the studies were clearly defined in advance to ensure transparency and reproducibility of the review. As with the definition of research questions in the social sciences, the SPICE framework (Booth, 2006; Petticrew and Roberts, 2006) was not used to determine the eligibility criteria for the scoping review. Although the SPICE framework guided the formulation of the research question, the eligibility criteria were defined based on the PCC framework, as recommended by Pollock et al. (2023). The PCC framework—Population, Concept, Context—is a widely used tool for developing research questions and defining eligibility criteria in scoping reviews. As outlined by Pollock et al. (ibid.), PCC provides a structured approach to clearly delineate the scope of a review, thereby enhancing the relevance and applicability of its findings. The population defines the target group or the group to be considered in the study. The concept specifies the topic of interest, and the context describes the setting, i.e., the framework in which the concept is examined.

The criteria for this scoping review are briefly described below.

- The population included in-service teachers actively engaged in classroom teaching, which corresponds to the third phase of teacher education. Trainee teachers and PLCs situated in university contexts were excluded from the review.
- The central concept focused on Professional Learning Communities (PLCs) in terms of their establishment, operational methods, and effectiveness. The groups that correspond to the description in the theory section are considered PLCs. Particular attention was given to the improvement of teaching practice through collaborative engagement and the co-construction of educational materials (high quality teaching is considered under the Opportunity-to-Learn-framework according to Vieluf et al. (2020) and Schmidt and Cogan (2015)). PLCs with a primary emphasis on internal processes rather than educational outcomes were not considered.
- The context was defined as the educational setting, with a particular emphasis on STEM (Science, Technology, Engineering, and Mathematics) education.

Beyond the inclusion and exclusion criteria outlined by the PCC Framework, this study also took the following aspects into account:

- This scoping study aims to examine the work of specific professional learning communities and gain a better understanding of their working methods and structure. For this reason, review articles and meta-analyses are excluded.
- To capture the broadest possible spectrum of literature on Professional Learning Communities (PLCs), this review included all publication formats, without restriction. This approach was taken due to the ongoing discussion regarding their conceptualization and systematization (Kansteiner et al., 2020; Bloh and Bloh, 2020; DuFour, 2004). The review therefore comprised peer-reviewed journal articles, book chapters, conference proceedings, dissertations, and relevant grey literature (e.g., reports from educational institutions or non-governmental organizations).
- Due to resource constraints as lack of translator availability as well as time limitations and to ensure accurate comprehension and interpretation, the review was limited to German and English language literature. We acknowledge that this may introduce a language bias.
- Given that PLCs emerged in the early 1990s and gained traction in Germany in the early 2000s, no restrictions were imposed on the publication year of the included literature. This approach ensures that the review captures the full historical development and evolution of PLCs.
- There are no exclusion criteria relating to geographical location.

All eligibility criteria mentioned here were taken into account both in the literature search and the filters applied, as well as in the subsequent title and abstract screening. This was partly carried out using AI, a large language model (LLM), which was also given these criteria.

### 2.2 Information sources

A wide range of information sources was consulted to ensure comprehensive coverage of the existing literature. These included bibliographic databases and subject-specific research portals. All sources were selected based on their subject relevance, accessibility, and completeness in terms of education-related and interdisciplinary content. The selection of information platforms and the research and screening within the databases took place between November and January 2025. In order to ensure comprehensive coverage, the literature was sourced from key educational databases. The *ERIC* (Educational Research Information Center) database was selected for its extensive collection of English-language educational research. Referred to ERIC- Website it provides premier bibliographic and full-text database focused on education research, globally accessible and maintained by the U. S. Department of Education’s Institute of Education Sciences (Institute of Education Sciences, 2025).

For German-language literature, the *Fachportal Pädagogik* was utilized, which incorporates the FIS (Specialized Information System for Education) database. The multidisciplinary search engine *BASE* (Bielefeld Academic Search Engine) -a multi-disciplinary search engine for academic web resources, not a traditional bibliographic database - was utilized to identify relevant open-access publications and grey literature. While broad databases such as Scopus or Web of Science were considered, the selection was focused on specialized educational databases in order to maintain relevance to the pedagogical context of this review. This focus is acknowledged as a potential limitation.

### 2.3 Search

In accordance with the PRISMA-ScR guidelines (Tricco et al., 2018), the electronic search strategy was developed to ensure transparency and reproducibility. For this review, an initial search was conducted in the Bielefeld Academic Search Engine (BASE), a multidisciplinary academic search engine that indexes open-access content from institutional repositories, academic journals, and digital libraries worldwide. The following example illustrates a search run in the BASE database.

The search was performed in November 2024. The results were limited to documents published between 1970 and 2025 and restricted to those in English. Initially, only keywords were used to assess the potential size of the sample before applying these filters. Over 200,000 entries were found for the term PLC alone. It was noted that the abbreviation of PLC is also used for *programmable logic controllers, public limited companies, and power line communication.* Therefore first, the language filters on BASE were set to English and German. In addition, BASE offers the option of filtering by Dewey Decimal Classification. Suitable classes would be DDC 371–374, but BASE only allows the selection 37*, which is summarized under *education and training*. This also includes the areas of higher education and education policy, which must be considered during the screening process. An overview of the search terms used can be found in the following Table 1.

**Table 1:** Search terms used in the BASE database.

Blocks	German	English	Boolean operators
Search terms	Professionelle Lerngemeinschaft	Professional Learning Community	OR; AND
	Professionelle Lerngemeinschaften	Professional Learning Communities	NOT (“programmable logic controllers,” “public limited companies,” and “power line communication”)
	PLG*	PLC*	* With truncation, to consider words with various endings (plural)

Final search string: (“Professionelle Lerngemeinschaft” OR “Professionelle Lerngemeinschaften” OR PLG* OR “Professional Learning Community” OR “Professional Learning Communities” OR PLC*) NOT (“programmable logic controllers” OR “public limited companies” OR “power line communication”).

Following the manual search, results were exported to an Excel spreadsheet for screening. During this preparation phase, duplicate entries and articles in incorrect languages were removed. A manual quality control was necessary to ensure the relevance of all entries, as the BASE interface does not support advanced export options.

### 2.4 Selection of sources of evidence

The review leveraged large language models (LLMs) to streamline the screening process. The models were selected based on two key criteria: their large context length and their proven capability to accurately assess input content in text comprehension tasks. This approach was taken to ensure the validity and precision of the results. At the time of the screening *Gemini Advanced 1.5 Pro* and *Gemini Advanced 2.5 Pro* were the best suited, commercially available models to the authors for this purpose (Team et al., 2024; Eagle, 2025).

Additionally, the authors employed consistent and thorough prompt engineering practices to increase the quality and reproducibility of the results. Among the prompt engineering practices used were few shot prompting (Brown et al., 2020) to improve accuracy in text comprehension. In addition, self-evaluation was employed, by generating not only screening decisions, but also decision justifications to further increase accuracy and allowed human reviewers to effectively assess plausibility and increases overall accuracy of the generated results by the LLM (Ren et al., 2023). Further quality improvements were achieved by generating a confidence score for all results, which not only aided reviewers, but again, is generally known to increase result accuracy in discrimination tasks (Tian et al., 2023). To ensure that relevant articles were not erroneously filtered or included, the screening results were systematically reviewed for consistency and plausibility. Manual corrections were performed were necessary, wherever the generated results were deemed inaccurate.

The implementation of these measures, namely systematic prompt engineering, the utilization of confidence scores, and the mandatory execution of manual reviews, collectively constituted a multifaceted quality assurance system. This approach was designed to optimize accuracy while ensuring that the final selection of evidence was made by the researcher.

### 2.5 Screening preparation

**Figure 1:**
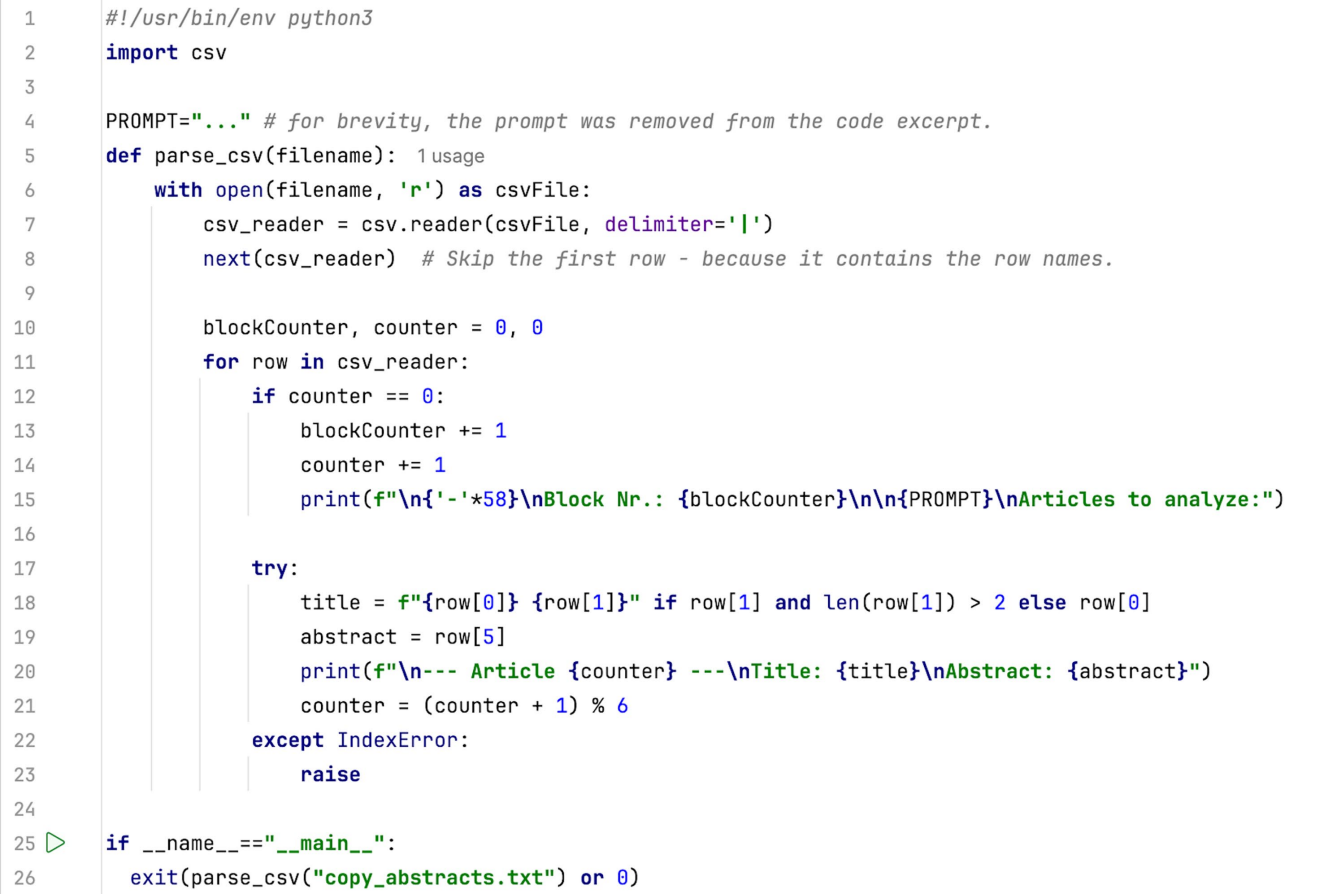
Python script to prepare abstract screening prompts from a CSV-formatted text file with a pipe delimiter (The title and subtitle were extracted from the first and second column, respectively. The abstract was extracted from the sixth column of the original data file. The abstracts were screened in groups of five; for each screening prompt, five articles were screened at once).

To prepare the data for systematic screening, contributions were exported from an Excel spreadsheet into a CSV file using a pipe delimiter (‘|’). This format enabled the automated generation of screening prompts via Python scripts (see Figure 1).

Although the Gemini Models employed have a large enough context length to potentially screen hundreds of titles and abstracts with only one well-crafted prompt, context confusion could affect screening results significantly (Liu et al., 2024). To balance accuracy and efficiency, titles were screened in groups of ten, abstracts in groups of five. A Python program was developed to format the data for pre-engineered prompts into appropriately sized groups. This was done to enable manual submission of the prompts via the common web interface, ensuring a systematic human review of the results rather than a programmatic API submission. Together with the engineered prompts, this made it possible to easily submit evaluation prompts which can be manually reviewed and checked for plausibility. The inclusion and exclusion criteria were included into the prompt, the LLM was instructed to indicate whether data sets should be included or excluded. In addition, a confidence value was to be specified, and a reason given for the inclusions and exclusions. The computed confidence value has proven especially useful, when checking the consistency of the screening, which will be explained later.

The combined approach of AI-supported extraction and manual control ensured that the efficiency of the screening was increased while also guaranteeing the quality and relevance of the selected sources. All decisions were documented in a transparent manner, and criteria for manual selection were defined in advance. This procedure is illustrated in the PRISMA flowchart, which visualizes the number of sources identified, screened, excluded, and ultimately included.

### 2.6 Screening process

The selection of sources for data extraction within 4,115 data sets was carried out in three steps. First, articles that were not in written in English or German, despite being specified by the filter were removed from the data, as well as the duplicates (*n =* 1,210). In addition, 61 articles were removed as records marked as ineligible by automation tools. The remaining articles (*n =* 2,844) were reviewed based on their titles and abstracts. In the third step, the full texts of potentially suitable sources were retrieved and evaluated based on the inclusion and exclusion criteria (see section 4).

The title screening was carried out after initial filtering was performed. The following few shot prompt was used to assist the reviewers with the title screening:

*“Filter and classify 10 scientific papers based on their titles and subtitles.*

*Criteria:*

*Inclusion: Titles or subtitles deal with professional learning communities (PLC, PLCs) in the context of school education.*

*Exclusion: Titles/subtitles have no connection to PLC in a didactic sense. Title/subtitle indicates a context outside of school/school education (e.g., medicine, crafts). Title/subtitle indicates that the paper is purely theoretical without empirical data.*

*Output: For each paper:*

*Title: [Title of paper]*

*Subtitle: [Subtitle of paper]*

*Classification: Include/Exclude*

*Confidence value: (0-10) - where 0 means “very uncertain” and 10 means “very certain.”*

*Reason for exclusion (if applicable): [Brief explanation of the exclusion in 1-2 sentences]*

*Example:*

*Input: 10 titles and subtitles of scientific works Output:*

*Title: Professional learning communities in mathematics education*

*Subtitle: An empirical study on promoting teacher competence*

*Classification: Include*

*Confidence value: 9*

*Reason for exclusion: -*

*Title: The importance of feedback in medical education*

*Subtitle: A qualitative analysis of case studies*

*Classification: Exclude*

*Confidence value: 10*

*Reason for exclusion: The title refers to the medical field and is therefore outside the school context. The study does not deal with professional learning communities. etc.* (for all 10 papers)

*Then, format the result into a CSV and output it again as a CSV, with title, subtitle, classification, confidence value, and reason for exclusion as columns.*

*## Papers for review:“*

**Figure 2:**
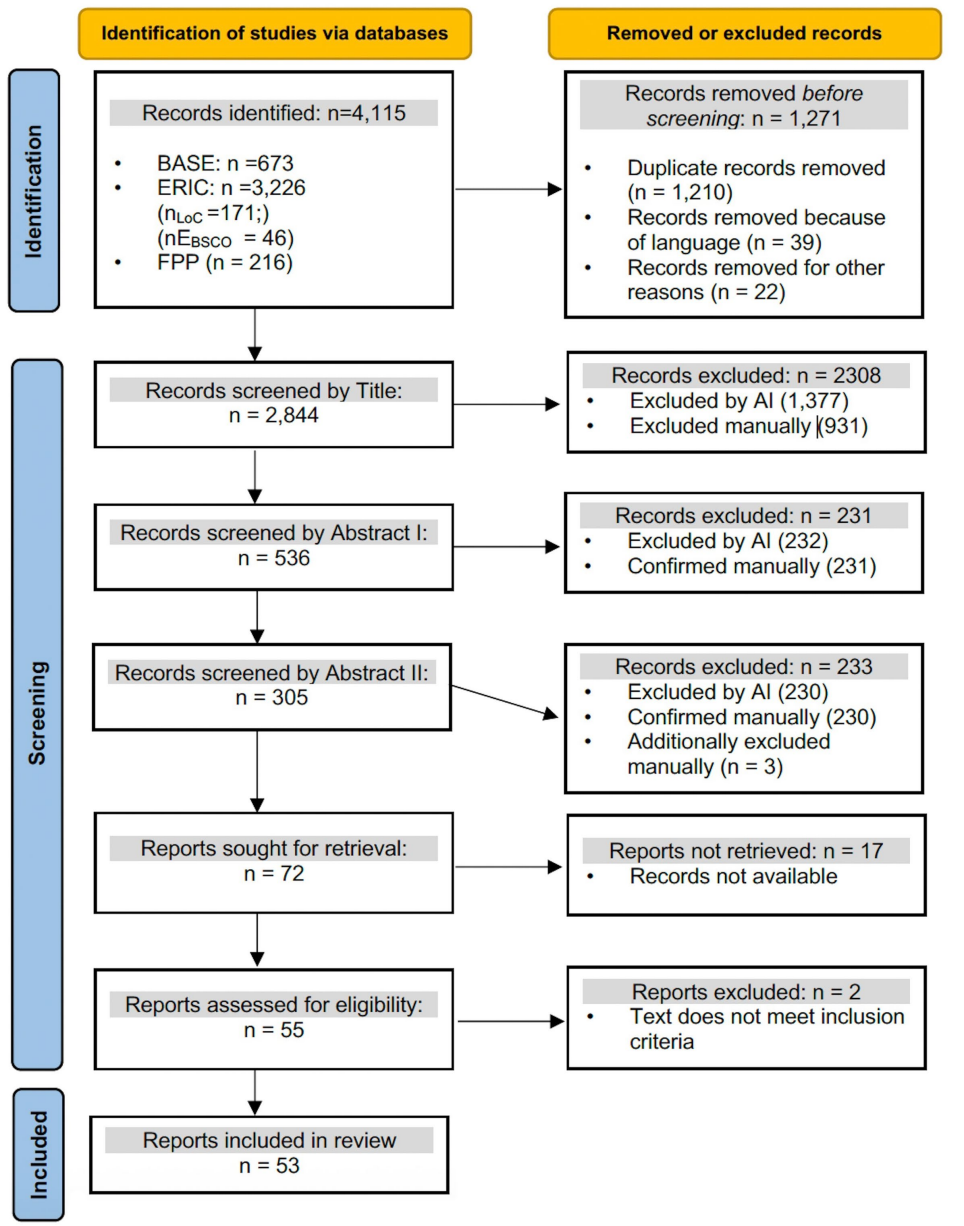
Flow diagram to show the selection of sources (framework adapted from Page et al., 2021).

After parsing and reviewing about half of the titles, we have determined this prompt to be effective when excluding articles. All excluded articles that Gemini excluded with a confidence score greater than 6, were accurate in the sense that a human reviewer has made the same decision. With exclusions with a confidence value under 6, the reviewers had to make some corrections. With inclusion, the AI was more generous using this prompt. This suggests that with this prompt, AI had higher sensitivity (finding potentially relevant articles) but lower specificity (incorrectly including more irrelevant articles) compared to manual screening. Manual screening of titles that were included by AI resulted in the exclusion of a significantly higher number of titles compared to the use of AI. The exact numbers can be seen in Figure 2 in the flow diagram. The reasons given by the AI for inclusions and exclusions suggest that the AI applies a clear structure to the selection process e.g.” *The title refers to heterogeneity and support in primary education, but not explicitly to PLC”* or “*This article describes a PLC situated within a university setting, focusing on faculty development for a principal preparation program, which falls outside the K-12 school focus.”*

Gemini was also used in the abstract screening process in a two-stage process. At the same time, manual screening was carried out for at least half of the contributions. During the abstract screening, it was again found that the prompt was accurate when excluding entries. When looking at the accuracy of inclusion, the prompt was again too generous. During the manual screening of 60% of the contributions and the quality of the inclusion reasons, we found that the AI favored inclusion over exclusion due to the wording of the prompt. The prompt was not clear enough about the fact that we considered some exclusion criteria as absolute, and that in some cases articles should be excluded regardless of any inclusion criteria met. In a second round of AI-assisted screening, the prompt was modified to require that the content of the contribution focus on the STEM field or the inclusion of digital tools or digitalization to ensure acceptance. Due to the higher complexity of the task, the prompt was engineered more carefully and is shared in Appendix 1. As a result of this modification, significantly more precise screening results were achieved in the second round of abstract screening.

### 2.7 Data charting process

An AI-supported process was also used to extract data from the articles. Before crafting a prompt, the authors sampled different types of articles that were screened and performed data charting manually. After gaining the necessary experience with the contents, a prompt was then crafted to assist the authors in extracting the relevant information from all articles. The relevant information from each included source extracted in a standardized format and then automatically transferred to an Excel spreadsheet. The authors then manually reviewed all entries to ensure their accuracy, completeness, and relevance. This manual check served as a quality assurance measure for the extracted data. A formal evaluation of the methodological bias of the included sources in the sense of a systematic quality rating was not performed, as this was not the focus of this scoping review.

To ensure a systematic data transfer after screening, a data charting form was developed in Microsoft Excel. This form was used to systematically extract relevant data from the included sources and can be found in full in Table 2. The form was designed to capture information directly related to the research questions of the review. The extracted data can be divided into four blocks:

- General information: Author(s), Year, Title, Subtitle, Language, Type of article, DOI (if available)
- Orientation of the article: STEM, Digitalization, Country, Theoretical framework
- Forms of PLCs: Composition, Size, Framing, Initiation, Workong method, Duration, Goal, Products
- Nature of the research: Methodology, Research approach, Data collection, Results

**Table 2:** Data items and anchor example for a dataset.

Category	Name	Definition	Options, if available	Anchor example
General information	ID-Mastersheet	The identification number from the master table corresponds to the number of the table containing all data that has been screened for scoping.	1–4,172	56
	Author(s)	People who wrote the article	–	Lücken, Markus
	Year	Year of publication	–	2012
	Title	Name of publication	–	Identification of characteristics of successful professional learning communities using the example of the “Biology in Context” (bik) project. (translated)
	Subtitle	Subtitle of the publication	–	–
	Language	language in which the article was published	German, English	German
	Type of article	Describes the type of text the publication has and where it was published.	Contribution to a collection, journal article, conference paper, monograph	Contribution to a collection
	DOI	Digital object Identifier	–	–
Orientation of the article	STEM field	Describes which area of STEM is covered in the article?	Biology, Mathematics, Physics, Chemistry, Computer Science, Integrated STEM, Other (multiple entries possible)	Biology
	Digitalization	Does the article focus on digitalization or the implementation of digital products or digital tools in the education sector?	Yes, No, Not reported	No
	Country	Describes in which country the PLC was active	–	Germany
	Theoretical Framework	Describes the theoretical basis or authors on which the work of the PLC or the article is based.	–	Bolam et al. Vescio et al.
Form of PLCs	Composition	Describes the groups of people that make up the PLC(s)	–	teachers from different schools, researchers, university staff, others
	Size*	Describes the size of the PLCs or the entire group, including the number of PLCs.	–	171 teachers in 11 PLCs.
	Framing	Describes the number of meetings, the duration of the meetings, and where the meetings took place.	–	Approx. 6 times per year (four half-day meetings and 2 two-day meetings)
	Initiation	Describes whose initiative led to the creation of the PLC. And if participation was voluntary	–	Initiated as part of the “Biologie im Kontext” project, funded by the German Federal Ministry of Education and Research (BMBF), voluntary participation.
	Working method	Describes how the PLC worked together (e.g., collaborative planning, co-construction, peer observation…)	–	Development of teaching materials, implementation and reflection of these materials, symbiotic cooperation between teachers, researchers, and administration representatives, reflection on PLC development using feedback forms.
	Duration	Describes how long the PLC has been working together in a coordinated manner	–	3 years
	Goal	Describes the purpose of the PLC	–	To support the professional development of biology teachers in developing, testing, and implementing competence- and context-oriented teaching units.
	Products	Describes which products were created in the PLC, if any	–	New tasks and teaching units for biology classes.
Nature of the research	Methodology	Describes how the research was conducted, i.e., which methods and procedures were used for data collection	–	Psychometric validation framework (including construct and prognostic validity).
	Research approach	Describes the research approach, i.e., the plan or strategy for data collection	Qualitative, quantitative, mixed method, not reported	Mixed methods
	Data collection	Nature of data that was collected	–	Questionnaires (for teachers and students), feedback forms, meeting protocols.
	Results	Describes concrete recommendations for action, conditions, obstacles, or considerations noted in the article (e.g., allocate dedicated meeting time, provide digital infrastructure)	–	Use the developed feedback instrument for formative evaluation and reflection within the PLC; Consider the six identified success factors (Cooperation, Shared Goal, Reflection, Outcome Orientation, Decision-making Freedom, Learning Attitude) in planning and coordinating projects; Future research should use more than two items for short scales and include objective measures of student competence.

*If an article contains a description of several PLCs, these are assigned letters in the data extraction sheet (e.g., 40a/40b, etc.), provided that they could be identified.

Where possible, the Excel table was provided with multiple choice options. This enabled uniform categorization, simplifying analysis and subsequent synthesis.

### 2.8 Synthesis of results

The synthesis was achieved through the implementation of a data charting process. A data charting form was utilized to organize the sources of evidence into key categories relevant to the objectives of the review. The synthesis itself involved a descriptive numerical summary to map the distribution of evidence across STEM research areas, publication years, and countries of origin. Furthermore, a qualitative content analysis was conducted to summarize the key concepts. The results obtained were structured in accordance with the research questions formulated and are presented in the form of a narrative summary and in tabular formats. To illustrate this point, the charting of characteristics of professional learning communities (PLCs) revealed significant variations in their reported size, composition, working methods, and outputs. During the thematic analysis of the extracted data, it became apparent that a significant proportion of studies had reported factors contributing to the effectiveness and success of PLCs. These factors were therefore established as a central, emergent theme for the synthesis and were charted and analyzed systematically. It is important to note that the ‘effectiveness’ or ‘success’ of a PLC was not a predefined inclusion or exclusion criterion. Instead, factors that the authors reported as contributing to the effective functioning of PLCs emerged as a key theme during the data charting and synthesis phase. This descriptive summary of the findings enabled the identification of key research gaps where evidence was found to be limited or inconsistent.

### 2.9 Selection of sources

The comprehensive literature search in the ERIC, BASE, and Fachportal Pädagogik databases yielded 4,115 relevant results. Following the removal of duplicates (*n =* 1,210), foreign-language entries (*n =* 39), and incomplete entries (*n =* 22), a total of 2,844 titles were subjected to further scrutiny. During the initial screening of titles, 2,308 articles were excluded, leaving 536 for abstract screening. At this stage, a further 464 articles were removed, resulting in 72 articles that were subjected to full-text analysis. Of these, 55 full texts were obtained. Following a thorough review of the literature, two articles deemed to be thematically irrelevant were excluded from the analysis, leaving a total of 53 studies for the final analysis. The sequence of events is illustrated in the flow diagram below.

In order to demonstrate the characteristics of the sources that have been included in the study, a list is provided in Appendix 2. In accordance with the objectives of this scoping review, no formal critical appraisal of the included sources of evidence will be conducted. The objective of this review is to furnish a thorough overview and mapping of the extant literature, as opposed to evaluating the methodological quality or risk of bias of specific sources. The subsequent section, therefore, pertains to the synthesis of the sources and the summary of evidence, wherein the four blocks are examined sequentially and summarized, culminating in the responses to the research questions.

## 3 Results

The ensuing sections present the synthesized findings from the included sources, structured according to the central themes that emerged from the analysis in relation to the research questions. Overall the analysis of the publications shows that the sample consists of 53 publications. These comprise eleven monographs (20.8%), four contributions to collected works (7.6%), two papers (3.8%) and 36 journal articles (67.9%) (rounded to one decimal places).

### 3.1 Summary of evidence

**Figure 3:**
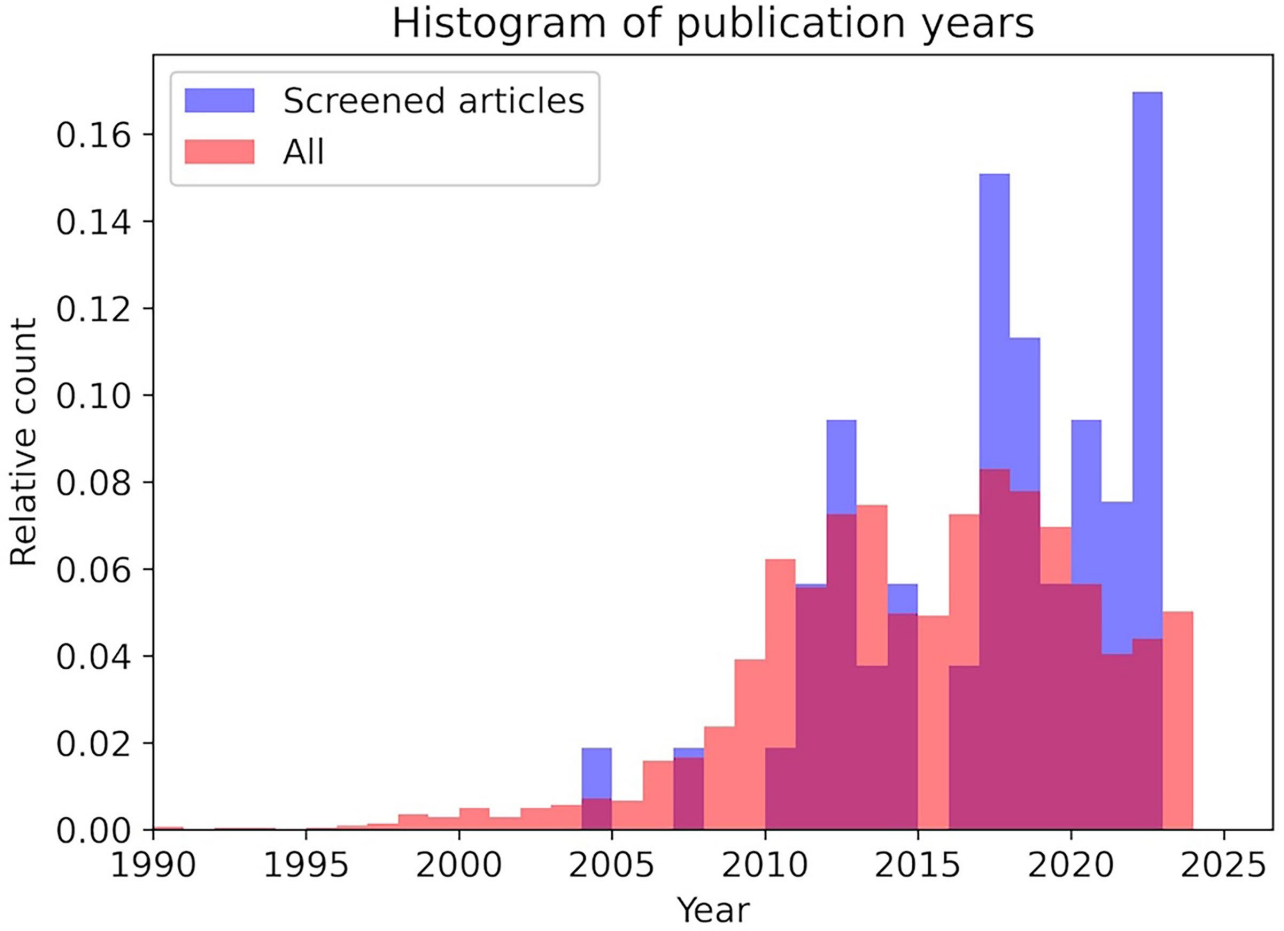
Histogram of all extracted publications across the years (red) and adjusted barplot of screened publications (blue) in relative count. The plot was cut off at 1990 to better visualize the comparison with the screened articles.

The majority of the publications were written in English, with three German publications being exceptions. Although publications are distributed between the years 2004 and 2023, a notable increase in research output is evident from 2010 onward. The temporal distribution of the included studies was consistent with the overall distribution of the retrieved records although there is a slight tendency toward newer publications in the selection which is consistent given the subject matter (Figure 3). This finding indicates that the screening process, incorporating both inclusion and exclusion criteria, did demonstrate a little systematic preference or toward publications from specific time periods. Even though, the included studies can be regarded as a broadly representative sample of the publication trends in the field because digitalization has only come into focus in the last decade (Table 3).

**Table 3:** Overview of STEM departments and focus of digitalization.

Department	Digitalization focus		Overall
	No	Yes	
Biology	2	2	4
Biology and Mathematics	0	1	1
Chemistry	0	1	1
Computer Science	0	2	2
Mathematics	7	10	17
Physics	1	0	1
Integrated STEM	7	5	12
Other	3	12	15
Overall	20	33	53

Most publications (*n =* 26) originate from the United States of America. A total of four publications were documented as originating from South Africa, while three publications were documented as having been published in Germany. It is noteworthy that no more than two articles originated from each of 13 other countries, including China, Thailand, the Netherlands, Philippines, Malaysia Bangladesh, India, Canada, Sweden, England, Isreal, Vietnam and Cameroon.

A clear trend toward mathematics can be seen when looking at the subject areas, as this subject accounted for almost a third of the publications with 17 contributions. Thirty-three of the 53 contributions also featured digitalization as part of the PLC’s work.

### 3.2 Theoretical references for PLCs

**Figure 4:**
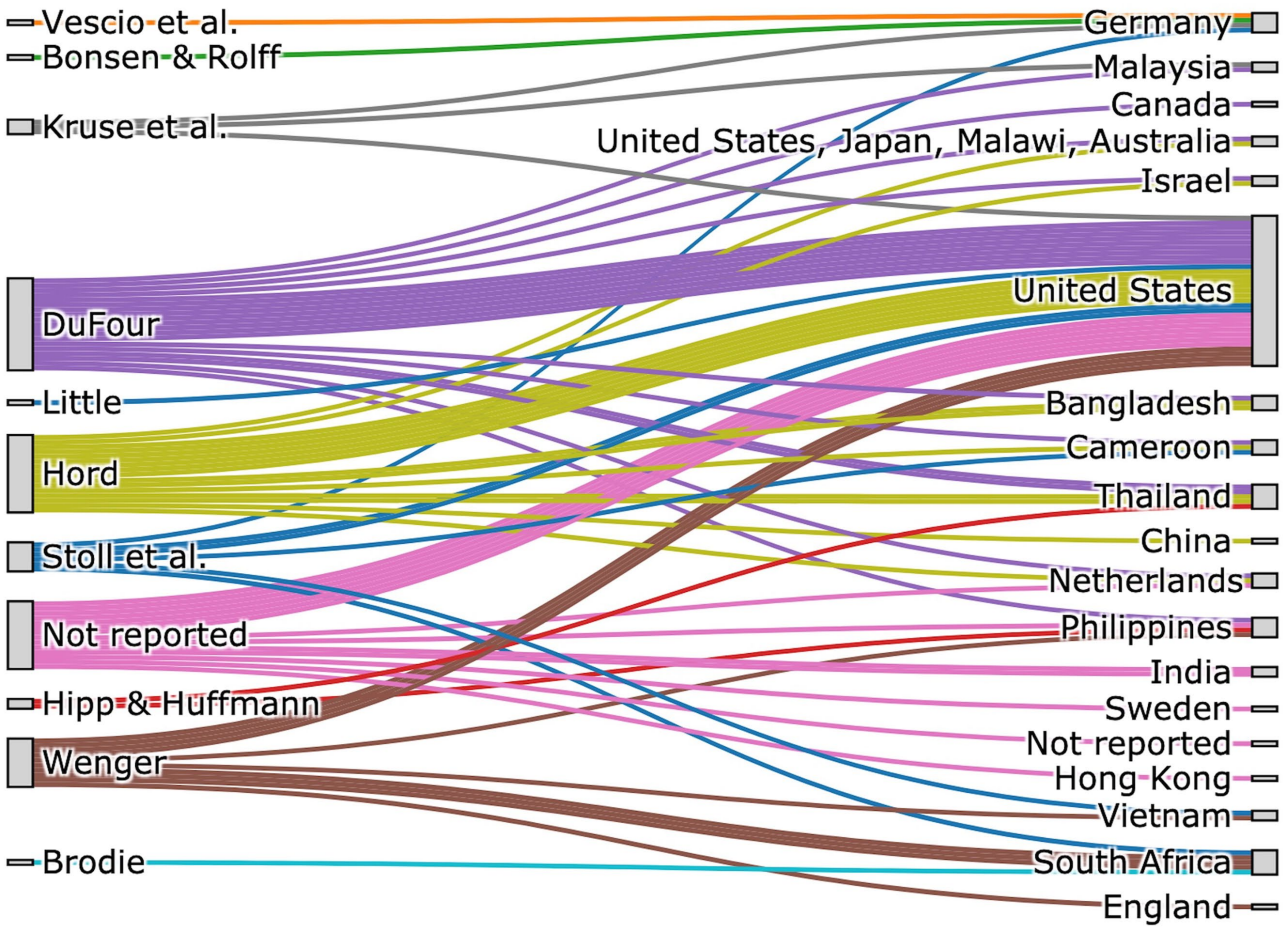
Sankey diagram showing the influence of different Author networks to the publications.

Due to the varying theoretical frameworks and emphases on PLC descriptions found in the literature, a textual analysis was conducted. This analysis aimed to determine the specific theoretical framework referenced in each report. Some publications also focused on other topics in their theoretical sections. This was noted as *Not reported* in the evaluation. The following Sankey diagram (Figure 4) shows the influence of the various works and authors on the publications. It is evident that many authors refer to the works Hord (1997) who, like Hipp and Huffman (2003, 2010) as well as Kruse and Johnson (2017) focus their work on descriptive models of professional learning communities. Many publications also focus on prescriptive implementation models developed by DuFour’s working group and summarized in the figure under the term “DuFour” (DuFour and Eaker, 1998; DuFour et al., 2008; DuFour, 2004). Authors from the US, South Africa, Vietnam, and the Philippines have also explored Wenger (1998) and Lave and Wenger (1991) theoretical foundations. Although he never explicitly refers to PLCs, he has used the concept of communities of practice and the underlying learning theories to explain why PLCs work. He sees communities of practice as an umbrella term, with PLCs then being an application in the field of education. The other authors referred to in the articles focus on specific topics in the context of PLC theory, such as research (see Vescio et al., 2008) or critical-analytical framework concepts (see Bonsen and Rolff, 2006; Stoll et al., 2006). The analysis indicates the presence of a distinct German-speaking discourse on PLCs, drawing on its own set of foundational texts (Bonsen and Rolff, 2006; Vescio et al., 2008) and appearing to be less connected to the dominant North American theoretical strands.

### 3.3 Structure of PLCs

The following chapter is divided into three sections. First, it discusses the composition of the PLCs from the various groups of people. Second, it covers the framing of the PLCs, i.e., the number and type of meetings, and the total duration of the PLC. Finally, it addresses the content dealt with by the PLCs.

#### 3.3.1 Composition and size of PLCs

The analysis of PLC composition was structured in two parts. First, the focus was on teachers as the central elements of the PLCs, followed by a consideration of any additional members. Of the 53 contributions, 25 PLCs have teachers from the same schools as members and 23 PLCs have teachers from different schools as members. In five PLCs, teachers from both the same school and different schools were represented. Furthermore, these PLCs can be grouped according to whether the teachers from the same schools worked with a facilitator (*n =* 8) or not. In the case of teachers from different schools, a distinction can be made between groups with a researcher and facilitator (*n =* 6) and groups with only a researcher (*n =* 17). The other components, such as the involvement of school management or external groups such as IT support or parents, cannot be clearly assigned.

It is challenging to provide a definitive statement or calculate the mean value of the size of PLCs, that is to say, the number of participating teachers. As indicated by the findings of certain publications, the number of participants has been reported to be 171, distributed across 11 PLCs, with analogous figures being reported in other cases. Consequently, the present analysis was conducted exclusively on the basis of publications that were able to provide a clear number, which corresponded to 44 publications. In instances where the numerical values ranged from 5 to 7, the mean value was assigned. This resulted in a minimum of two teachers as members of a PLC (M = 9.27; SD = 9.28; Md = 6). The substantial standard deviation, which closely resembles the mean, signifies a considerable degree of variability in PLC size, extending from diminutive, intimate groups to substantially larger collectives.

#### 3.3.2 Framing of the PLCs

**Figure 5:**
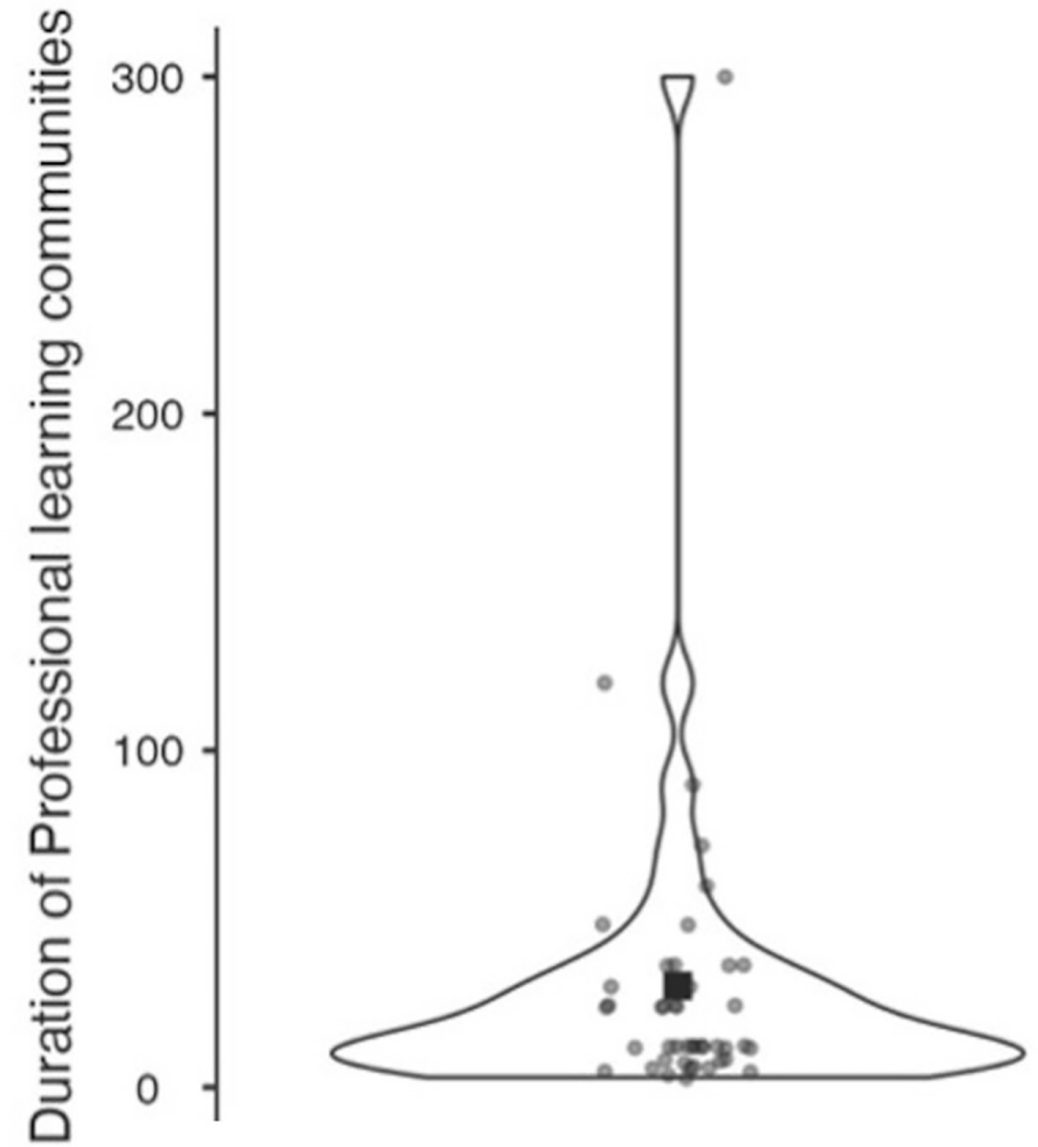
Violin-Plot showing duration of PLC work in months.

When it comes to personnel and organizational structures, the duration of the of the PLCs varies as it can be seen in Figure 5. If a group specified a time period, the mean value was used. With a few exceptions, PLCs have a maximum lifetime of two years. The median is twelve months. The upper quartile value is 31.5 months, or 2.5 years. There are a few exceptions (e.g., ID:21) that have existed for more than five years.

A comprehensive analysis of a substantial number of documented cases of collaboration in professional learning communities (PLCs) reveals considerable heterogeneity in cooperation models. There is no universally applicable standard; instead, structures adapt to the respective contextual conditions. The range of approaches can be systematized according to the following dimensions: frequency, duration, modality, and structural anchoring.

A distinguishing feature is the frequency of collaboration. The spectrum ranges from very intensive to sporadic models. In the sample studied, at least five models were based on daily meetings lasting between 30 and 45 min, or developed from other frequencies into daily short visits. The most frequently mentioned frequency was weekly meetings, which were documented in at least eleven cases and often lasted one to two hours. In addition to more intensive formats, less intensive yet regular formats were employed, including biweekly, monthly, or bimonthly meetings. A further category comprises event-based models, within which cooperation is concentrated on a select number of time-consuming appointments. These include several full-day workshops per year, week-long summer institutes, or a predetermined number of meetings lasting several hours per semester. The organization of meetings in teachers’ working days is closely linked to frequency. It is evident that numerous PLCs have integrated collaboration into the school day, employing various strategies such as joint planning periods, the final lesson of the day, or designated time slots. Conversely, a significant number of meetings were scheduled outside of standard teaching hours, including after school, in the evenings, and during weekends and summer holidays. In conclusion, the models demonstrate a divergence in their degree of structure. In addition to scheduled, regular meetings, there are also highly flexible approaches. In certain contexts, *ad hoc* time for collaboration was allocated, or the frequency of collaboration varied depending on necessity. The cooperative endeavor encompassed both formal meetings and informal, spontaneous conversations in corridors or classrooms. In certain models, a process-oriented development was also observed, characterized by a shift in structure over time. This transition manifested through transitions from monthly observations to daily reflections or from weekly to daily meetings. A striking finding in the evaluation is that in a significant number of cases – in at least 13 of the descriptions examined – essential information such as the exact frequency or duration of the collaboration was not reported. This finding suggests a potential lacuna in the current documentation practices, thereby hindering the execution of a comparative analysis that would evaluate the efficacy of diverse cooperation models.

#### 3.3.3 Work processes within PLCs

The endeavors undertaken within PLCs are typified by the close interaction between three central working methods, which seldom occur in isolation. The most frequently described working method is joint reflection, which is explicitly mentioned in 48 of the 53 sources (91%). This encompasses, in particular, data-supported analysis of student work, teaching videos, and learning outcomes. For example, as described in Stegmann et al. (2022a, 2022b), the process often involves teachers presenting their lessons to each other, sharing materials, and providing mutual suggestions for improvement, using the shared resources as a foundation for collaborative redevelopment. The second most prevalent category is co-construction, defined as the collaborative development of lesson plans, materials, or assessment instruments, which is documented in 43 sources (81%). The findings reveal that supervision, predominantly in the form of peer observation or co-teaching, is present in 32 of the 53 sources (60%). The integration of these working methods is a key finding, with 24 sources (45%) describing activities from all three categories as a coherent cycle. A further 18 sources (34%) demonstrate a close link between co-construction and joint reflection. A synthesis of the extant literature reveals that 79% of the examined studies describe a process where at least joint planning or creation is followed by reflection. Isolated working methods are the exception to this; only one source described supervision or reflection as the sole activity. The numerical analysis thus confirms that the typical PLC process is an integrated cycle that combines collaborative creation, collegial observation, and data-supported reflection to improve teaching practice.

### 3.4 Research into PLCs

An analysis of the methodological approaches used in the 53 studies included in the review reveals a clear predominance of qualitative research designs in the investigation of PLCs. A total of 41 studies (77.4%) used a qualitative approach, while only two studies (3.8%) were purely quantitative and three studies (5.7%) were designed as mixed-methods studies. Within the field of qualitative research, case studies were the most frequently used approach (*n =* 14). This was followed by approaches such as grounded theory and action research. Regarding to data collection, the studies analyzed consistently pursued an approach that combined various methods. The most frequently used instruments for data collection in this context are interviews in various formats (individual or group), observation of PLC meetings and teaching units, and analysis of documents and artifacts. Video and audio recordings are frequently used to support observations and for detailed analysis of interaction processes. Questionnaires and surveys, often in combination with Likert scales and open-ended questions, are also used for standardized data collection. The systematic combination of various data sources presented here suggests that PLCs are almost exclusively studied in a multidimensional way. Instead of relying on a single data source, studies use a form of triangulation to obtain a comprehensive picture of the research subject. A typical approach, for example, combines online surveys, interviews, document analyses, and observations within a single study. This practice allows the complexity of PLCs to be captured. To this end, the perspectives of the participants (through interviews and questionnaires) are related to their actual practices (through observations) and the resulting work outcomes (through artifact analysis).

In order to organize the diverse success factors from the literature in a systematic manner, the 112 extracted recommendations and research findings were subjected to a thematic categorization and quantitative analysis. The analysis reveals that the factors contributing to success are concentrated around a small number of main themes, which are found to occur with high frequency. Table 4 provides a comprehensive overview of the distribution of mentions across the identified main categories.

**Table 4:** Thematic focus of success factors for PLCs.

Focus	Number of mentions
Resources (structural and material)	39
Team culture and collaboration	28
Content and process design	24
Leadership and administrative support	23
Role of facilitator	10
Recommendations for future research	6
External support and partnerships	4

As demonstrated in the table, structural and material resources (*n =* 39) represent the most frequently mentioned thematic area by a significant margin. Within this category, time emerges as the most critical single resource, explicitly cited as a crucial prerequisite for successful collaboration in 17 of the analyzed sources. This is closely followed by the culture of collaboration (*n =* 28), which encompasses aspects such as trust, a shared vision, and psychological safety. Additionally, the content and process design of the PLC’s work (*n =* 24) — that is, the activities undertaken by the team and the methods employed — and the active support from leadership and administration (*n =* 23) were found to be of high relevance. The reviewed studies illustrate that this support manifests in various, complementary forms of leadership. On an organizational level, effective leadership involves concrete actions by the school administration to provide structural resources. For example, Stegmann et al. (2022a, 2022b) describe how principals actively creatie protected time slots for PLC-work within the regular working hours, which signals the value placed on these activities. On a visionary level, leadership provides a guiding purpose that inspires collaborative work. This support often manifests as a guiding vision. Wong (2010), for example, identifies a leader’s vision “renew the existing culture and system*”* (p.137) as a key factor for the sustainability of a PLC. Finally, leadership also emerges from within the team through expertise and knowledge. Woolway et al. (2019) demonstrate that the success of a PLC, particularly in its initial stages, is “hugely dependent on at least one person in the group taking the lead”(p.11) who possesses strong pedagogical and content knowledge.

While less frequently mentioned, the specific role of the facilitator (*n =* 10) and the importance of external support (*n =* 4) are still identified as relevant. A small group of studies (*n =* 6) also formulated explicit recommendations for future research, primarily calling for the use of experimental designs and the investigation of long-term effects.

## 4 Discussion

The objective of this scoping review was to systematically map the landscape of Professional Learning Communities in the STEM field and in the creation of digital products. The findings were then used to derive guidelines for designing a PLC framework fostering teachers’ digital readiness in out-of-school learning. The analysis of 53 relevant publications reveals a heterogeneous and, in some respects, limited research landscape, as already described by Kansteiner et al. (2020), but nevertheless it also provides clear implications for practice.

### 4.1 Summary and key findings

Four key findings serve as the basis for the following discussion. First, the existing body of research exhibits significant limitations. The literature is dominated by studies from the United States focusing on mathematics. Furthermore, qualitative, descriptive case studies predominate from a methodological perspective, resulting in a scarcity of generalizable, causal evidence regarding the impact of PLCs on teacher practice and student outcomes. Second, the structural characteristics of PLCs are highly diverse, suggesting that no single model fits all contexts. Third, despite this structural diversity, a common processual core was identified which indicates a shared understanding of how professional development within a PLC should function. Finally, a key emergent finding was the consistent identification enabling for collaboration. Across different contexts and PLCs, structural resources and supportive leadership were the most frequently cited prerequisites for the working of these communities. These findings highlight the diverse structures, common processes and notable gaps within the research landscape. They will be analyzed and discussed in subsequent sections to derive implications for practice and future research.

### 4.2 Critical analysis of the PLC research landscape

A salient finding of this review is the restricted generalizability of extant research. The majority of extant studies originate from the USA and focus on mathematics. The findings’ transferability is limited by their narrow geographical and disciplinary focus. This makes their application to other subjects and particularly to different educational contexts, such as those in Europe and Germany, challenging. The prevailing focus of research on the US context and the subject of mathematics means that the dominant models and success factors for PLCs are embedded in a specific educational policy and school culture framework. This considerably restricts their direct applicability to the German school system and other STEM subjects, emphasizing the necessity for context-sensitive adaptation. Moreover, the primary focus of the publications is on descriptive-analytical accounts of PLCs. In the field of research on PLCs, the majority of articles concentrate on quality. In this instance, too, it is challenging to generalize the results. In order to investigate the effectiveness of these systems, there is an urgent need for a research tool that can be applied to all possible forms of PLCs. Although there are few quantitative studies in this field, the existing ones systematically evaluate the effectiveness of the aforementioned phenomena. This highlights a research gap in terms of reliable evidence for the effectiveness of PLCs, which has already been clearly described by Warwas and Schadt (2020). The extant evidence base is dominated by qualitative case-specific studies, which further reinforces the situation already criticized by Vescio et al. (2008) that there is little reliable evidence available on the effectiveness of PLCs on student performance. The field of research is characterized by a plethora of detailed descriptions of the process dynamics of individual groups. However, there is a paucity of general, causal evidence of effectiveness. This discrepancy underscores the imperative for the expeditious development of a uniform research instrument.

### 4.3 Implications for implementing a PLC

The PLCs reviewed significant diversity in terms of size and duration. An optimal duration is difficult to determine, partly because many PLCs are initiated externally by researchers or universities, and their existence often depends on limited project funding. This diverse nature of PLCs in comparable professional fields indicates that a single, standardized model is inadequate for creating one’s own effective PLC. The adopted approach must be sufficiently flexible to accommodate the diverse needs of the participants. This is especially crucial for group size, meeting frequency, and meeting duration. In our context, it is imperative to favor intensive and flexible co-construction over impromptu interactions, as this approach is essential for the successful development of digitally enhanced learning opportunities in out-of-school settings. A desirable goal should be the sustainable continuation of the PLC independent of external support. Ideally, a PLC should be able to function independently following the provision of support by researchers. However, this is not evident in publications, which are predominantly authored by researchers themselves.

A consistent feature is the composition of the groups. PLCs rarely consist exclusively of teachers. Typically, the process is initiated, supports and organizes by researchers or facilitators such as coaches or multipliers or both. Based on their compositions and the nature of support PLCs can be grouped into two different clusters, representing one possible approach to their categorization.

- At a process level: The nature of the cooperation is contingent upon the PLC’s objectives, therefore a shared understanding of goals and collaboration models is important. For developing products, as intended in our project, co-construction appears to be a logical and effective working method.
- At a resource level: The most frequent factors for success are Resources (structural and material), particularly time. While a collaborative culture is considered important, the greatest challenge lies in the pragmatic and organizational integration of a PLC’s work into daily school life, which is highly dependent on support from school administration.

The findings of this overview indicate that effective PLCs extend beyond the mere exchange of materials or coordination based on the division of labor. In 81% of the studies examined, co-construction is described as the central form of work, meaning that work in PLCs can be characterized as the highest form of teacher cooperation (Grosche et al., 2020). In contradistinction to less complex forms of cooperation, co-construction focuses on the collective generation of new knowledge and new artefacts (e.g., teaching concepts). This process necessitates a high level of engagement and mutual dependence on the part of the participants. However, as research indicates, this exacting form of cooperation is associated with specific conditions for success that are challenging to fulfil.

This review’s analysis of PLC structures can be interpreted against the backdrop of the central challenges of co-construction. Grosche et al. (2020) identify the autonomy-parity dilemma as a core issue in intensive cooperation. This describes the tension between teachers’ desire for professional autonomy (self-determination in their actions) and the need for parity (equality and consensus-building) within the group. The findings regarding success factors can be directly interpreted as mechanisms for addressing this dilemma.

- The utilization of resources and the presence of effective leadership serve as the foundational framework for the organization. The most frequently cited success factor in the extant literature – namely, resources, especially time and support from school administrators – is not only an organizational requirement but also a structural prerequisite for mitigating the dilemma. This finding also strongly aligns with the foundational frameworks of PLCs discussed by Hord (1997) and Stoll et al. (2006), who both identify supportive conditions and supportive leadership as essential structural pillars. Our Review confirms that without this institutional backing, even the most willing teachers struggle to establish a sustainable collaborative culture. The allocation of adequate time is indicative of appreciation and serves to mitigate the pressure that arises when cooperation must be conducted surreptitiously. The efficacy of school management in this regard is of particular significance, as it can serve to legitimize the outcomes of PLCs, thereby enhancing the autonomy of teachers as individuals, and counteracting any potential erosion of that autonomy.
- Hierarchical structures and external moderation: The finding that PLCs are often accompanied by researchers or external moderators can be interpreted as an attempt to structure cooperation processes. Recent findings have indicated that this approach enhances efficiency. However, it should be noted that there is a concomitant risk of compromising the equity of participants, a prerequisite for achieving parity (Grosche et al., 2020). Consequently, effective facilitation must endeavor to empower the group to self-organize, as opposed to the imposition of solutions from the facilitator.
- The importance of cooperation in achieving success: The second most salient finding is that a robust culture of cooperation, predicated on trust and psychological safety, is the direct key to productively resolving the autonomy-parity dilemma. It is only within an environment characterized by trust that educators are willing to relinquish their autonomy in the pursuit of a collective objective, placing their reliance on the expertise of their peers. In the absence of this foundational element, co-construction rapidly devolves into a superficial exchange or enforced conformity.

### 4.4 Implications for further research

While this scoping review provides a comprehensive overview of the existing literature, it also highlights several significant gaps and unanswered questions. To advance the field, future research should priorities the following areas. Primarily, the emphasis on the US-American context and mathematics restricts the generalizability of the present findings. There is an urgent requirement for studies to be conducted that investigate the role of PLCs in diverse educational systems, particularly in Europe, and across a more extensive range of STEM subjects, including biology, chemistry, and engineering, in order to comprehend the context-specific success factors. The present review also corroborates the critique proposed by Vescio et al. (2008), validating an enduring paucity of substantial quantitative evidence. It is recommended that future research employ quasi-experimental designs in order to establish a causal relationship between PLC participation and measurable outcomes. Such a relationship should be investigated not only in teaching practice but also in student performance and engagement.

The call for a uniform research instrument remains highly relevant. A significant methodological challenge in PLC research is the absence of standardized measurement instruments, which hinders the capacity to compare and synthesize results across studies. The development and rigorous validation of a uniform instrument is therefore considered a key task for future research projects. A tool of this nature should be designed to systematically capture not only the process characteristics of PLCs – such as the quality of collaborative discourse, the degree of co-construction, and the type of facilitation – but also the outcomes at multiple levels, including changes in teacher practice and student learning. The availability of such a validated instrument would represent a significant step toward establishing a cumulative knowledge base and enable reliable comparisons that demonstrate which PLC models are most effective.

### 4.5 Limitations

The primary risk is attributable to restrictions in the selection of databases and the limitation to publications in specific languages. It is acknowledged that the exclusion of broad multidisciplinary databases such as Scopus and Web of Science may have resulted in the omission of some relevant articles from adjacent fields. Nevertheless, our meticulous approach to core educational databases, in conjunction with a meticulous search for grey literature, furnishes a comprehensive mapping of the primary research landscape. Despite the thorough nature of the database searches conducted, it is possible that relevant studies were not identified due to the exclusion of non-English or non-German articles, or limited access to certain databases. Consequently, the extant evidence base may not fully capture all existing research on the topic. Of the 71 publications identified, 17 could not be retrieved in full text. Access was attempted through multiple institutional and university library subscriptions, interlibrary loan requests, and direct personal contact with the authors via email and ResearchGate. Despite these efforts, no full texts had been obtained by the time of data extraction. Purchasing these items would have incurred costs, which was deemed disproportionate given the scope and resources of this scoping review. The unavailable works consisted predominantly of monographs and only two journal articles. Given that monographs often serve as supplementary background literature rather than core peer-reviewed sources in this field, the exclusion of these works is likely to have a limited impact on the overall evidence base, which remains robust with most journal articles included. Nevertheless, these studies were excluded from the full-text analysis but, where available, their abstracts or bibliographic summaries were screened to assess potential relevance. This exclusion is acknowledged as a potential source of bias. Where possible, information from abstracts or summaries has been integrated to minimize the impact on the breadth of the review. It is conceivable that this slightly impairs the complete picture of the research field.

## Data Availability

The original contributions presented in the study are included in the article/supplementary material, further inquiries can be directed to the corresponding author.
