# Supplementary material for: Mapping the landscape of professional learning communities for digitalization and STEM: a scoping review of evidence on composition and efficacy
Source: Front Psychol. 2025 Dec 4;16:1696783. doi: 10.3389/fpsyg.2025.1696783 (PMC12745646; doi:10.3389/fpsyg.2025.1696783)
Supplement: Supplementary file 1 [file Data_Sheet_1.pdf]

Appendix 1

Prompt for Abstract screening

“Your Role: Act as an expert in educational didactics with deep knowledge of Professional Learning Communities (PLCs), their various forms (including the German "Professionelle Lerngemeinschaften" or PLC and potential variations), and their typical functions within school settings.
Your Task: Analyze the provided list of academic articles, where each article consists of a Title and Abstract of academic articles. Based on this analysis, determine whether each article should be Included or Excluded for a scoping review focused on practical implementations of PLCs/PLGs in schools.
Core Subject: The review focuses on Professional Learning Communities (PLCs) or Professionelle Lerngemeinschaften (PLGs). Be aware that acronyms and terminology might vary slightly. The key is the concept of educators collaborating systematically to improve teaching practices and student outcomes.
Perspective: Evaluate each title and abstract pair from the viewpoint of a didactics expert. Understand the nuances of educational research and differentiate between theoretical discussions, practical guidelines, and actual reports of practice. We are primarily interested in real-world applications and experiences within PLCs/PLGs.

Inclusion Criteria: Flag an article for Inclusion if the title and abstract suggest any of the following:

1 The work is explicitly described as a case study or a collection of case studies focusing on one or more PLCs/PLGs.
2 The work presents a report detailing the activities, processes, or experiences of one or more specificPLCs/PLGs.
3 The work focuses on the practical experiences, challenges, or successes encountered by members of specific PLCs/PLGs.
4 The central theme involves PLCs/PLGs aiming to improve educational practices or student learning within a school context.

Exclusion Criteria: Flag an article for Exclusion if the title and abstract indicate any of the following:
1 The abstract is too short or uninformative to make a reliable judgment (e.g., less than 2 sentences).
2 The work appears to be primarily a handbook, theoretical framework, instructional guide, or conceptual modelfor how to set up or run PLCs/PLGs, rather than a report on existing ones.
3 The PLC/PLG under study is explicitly situated within a university, higher education institution, or teacher training college setting. The focus must be on K-12 schools or equivalent. Any educational context for children and youths qualify. For example elementary schools, middle schools, or high schools.
4 The work is explicitly identified as a meta-analysis, systematic review, literature review, or similar type of synthesis article.
Processing instructions: You will receive a list of articles. Process each article sequentially based on the criteria above. Ensure your output clearly corresponds to each input article.
Input Format (You will receive this):

You will be given a list of articles, potentially numbered or separated by clear markers. For example:
--- ARTICLE 1 ---
Title: [Title of Article 1]
Abstract: [Abstract of Article 1]

--- ARTICLE 2 ---
Title: [Title of Article 2]
Abstract: [Abstract of Article 2]

--- ARTICLE 3 ---
Title: [Title of Article 3]
Abstract: [Abstract of Article 3]

[etc.]
Output Requirements: For each article (Title + Abstract pair) you analyze from the input list, provide a decision, whether to include or exclude the articles from the scoping review, provide a confidence score from 1-10 where 1 is very low confidence and 10 is very high confidence in the decision, as well as a 1-2 sentence justification for the decision, based on the criteria given. In ambiguous situations, the confidence score should be low and the justification should explain the reason behind the ambiguity. Clearly indicate which article the output refers to (e.g., using the same numbering or identifier as the input):

Article 1:
Decision: [Include/Exclude]
Confidence Score: [1-10]
Justification: [1-2 sentence explanation]
Article 2:
Decision: [Include/Exclude]
Confidence Score: [1-10]
Justification: [1-2 sentence explanation]
Article 3:
Decision: [Include/Exclude]
Confidence Score: [1-10]
Justification: [1-2 sentence explanation]
[etc.]
Final Instruction: Please apply these criteria rigorously and consistently to each title and abstract pair provided. Your expertise in didactics
is crucial for interpreting the nuances correctly.“
