# Supplementary material for: Mapping the landscape of professional learning communities for digitalization and STEM: a scoping review of evidence on composition and efficacy
Source: Front Psychol. 2025 Dec 4;16:1696783. doi: 10.3389/fpsyg.2025.1696783 (PMC12745646; doi:10.3389/fpsyg.2025.1696783)
Supplement: Supplementary file 2 [file Data_Sheet_2.pdf]

Appendix 2

Publications included in the analysis in alphabetical order showing the characteristics of each source of evidence

Author	Year	Title	Language	Type of Article	DOI/Link
Anderson, Robin Keturah; Williams, Martia	2023	Mathematics Teachers´ Participatory Patterns between Face-to-Face and Virtual Professional Learning Environments	english	journal article	https://eric.ed.gov/?id=EJ1380375
Baricaua Gutierez, Sally	2016	Building a Classroom-Based Professional Learning Community through Lesson Study: Insights from Elementary School Science Teachers	english	journal article	http://dx.doi.org/10.1080/19415257.2015.1119709
Barno, Erin; Dietiker, Leslie	2022	Collective Curricular Noticing within a Mathematics Professional Learning Community	english	paper	https://files.eric.ed.gov/fulltext/ED630355.pdf
Berglund, Helena	2022	Biology teachers; collaborative experiences: benefits and difficulties in different contexts in relation to perceived value.	english	journal article	https://doi.org/10.1007/s11422-022-10127-2
Bond, John Kenneth	2019	Effects of Professional Learning Communities on Instructional Revisions in Secondary Mathematics Classrooms	english	monograph	https://mds.marshall.edu/cgi/viewcontent.cgi?article=2226&context=etd
Bonsen, Martin; Hübner, Carola	2012	Unterrichtsentwicklung in Professionellen Lerngemeinschaften	german	contribution to a collection	https://www.fachportal-paedagogik.de/literatur/vollanzeige.html?FId=3156095
Brendefur, Jonathan L.; Whitney, Brian; Stewart, Roger A.; Pfiester, Joshua; Zarbinisky, Julia	2014	Instructional Learning Teams: A Case Study	english	journal article	https://doi.org/10.5430/jct.v3n1p36
Brig, Leane	2014	How I Learned the Value of a True PLC	english	journal article	https://kappanonline.org/learned-value-true-plc-leane/
Brodie, Karin; Chimhande, Tinoda	2020	Teacher Talk in Professional Learning Communities	english	journal article	https://files.eric.ed.gov/fulltext/EJ1255537.pdf
Casas, Martin	2019	Professional Learning Community (PLC) Autonomy & Trust -- A Cross Case Study	english	monograph	https://escholarship.org/uc/item/3fd0s8mv
Chauraya, Million; Brodie, Karin	2017	Learning in Professional Learning Communities: Shifts in Mathematics Teachers´ Practices	english	journal article	https://doi.org/10.1080/0035919X.2017.1350531
Chauraya, Million; Brodie, Karin	2018	Conversations in a professional learning community: An analysis of teacher learning opportunities in mathematics	english	journal article	https://doi.org/10.4102/pythagoras.v39i1.363
Cheng, Pamela	2017	Professional Learning Community (PLC): Technology Integration at a Title I Elementary School	english	monograph	https://doi.org/10.31979/etd.58wj-dk5q
Dalby, Diane	2021	Professional Learning through Collaborative Research in Mathematics	english	journal article	http://dx.doi.org/10.1080/19415257.2019.1665571
Durr, Tony; Kampmann, Jennifer; Hales, Patrick; Browning, Larry	2020	Lessons Learned from Online PLCs of Rural STEM Teachers.	english	journal article	https://doi.org/10.35608/ruraled.v41i1.555
Francis, Donald Spencer	2011	Improving a Professional Learning Community at One Elementary School: An Action Research Study	english	monograph	https://citeseerx.ist.psu.edu/document?repid=rep1&type=pdf&doi=1a394f2821e03fd3690b3b3c6b329a095ca0f65f
Franks, Douglas	2012	Mathematics Professional Learning Communities: Opportunities and Challenges in an Elementary School Context	english	contribution to a collection	https://slub.qucosa.de/api/qucosa%3A1708/attachment/ATT-0/
Hardman, Elizabeth L.	2012	Supporting Professional Development in Special Education with Web-Based Professional Learning Communities: New Possibilities with Web 2.0	english	journal article	http://doi.org/10.1177/016264341202700402
Harris, Emily; Rosenman, Amelia	2017	Discussing Science in Professional Learning Communities	english	journal article	https://eric.ed.gov/?id=EJ1155391
Higgs-Horwell, Melissa; Schwelik, Jennifer	2007	Building a Professional Learning Community: Getting a Large Return on a Small Investment--I Get by with a Little Help from My Friends	english	journal article	https://eric.ed.gov/?id=EJ779149
Hillman, Peter Charles	2018	Vertically Aligned Professional Learning Communities as a Keystone for Elementary Science Teacher Professional Development, Growth, and Support.	english	monograph	https://doi.org/10.7916/D82N6JQH
Housen, Monica	2018	Disrupting High School Students' Mathematical Disaffection through a Professional Learning Community: A Retrospective Case Study	english	monograph	https://hdl.handle.net/2047/D20316426
Huggins, Kristin Shawn; Scheurich, James Joseph; Morgan, James R.	2011	Professional Learning Communities as a Leadership Strategy to Drive Math Success in an Urban High School Serving Diverse, Low-Income Students: A Case Study	english	journal article	https://doi.org/10.1080/10824669.2011.560525
Jones, Marvin B.	2018	An Exploration of Teachers' Lived Experiences in Professional Learning Communities in One Ohio Urban School	english	monograph	https://ecommons.udayton.edu/graduate_theses/6684/
Kasinathan, Gurumurthy; Ranganathan, Sriranjani	2017	Teacher professional learning communities: A collaborative OER adoption approach in Karnataka, India (Advance online publication)	english	monograph	https://doi.org/10.5281/zenodo.160680
Kloser, Matthew; Edelman, Amanda; Floyd, Catherine; Martínez, Jose Felipe; Stecher, Brian; Srinivasan, Jayashri; Lavin, Erin	2021	Interrogating Practice or Show and Tell?: Using a Digital Portfolio to Anchor a Professional Learning Community of Science Teachers	english	journal article	http://dx.doi.org/10.1080/1046560X.2020.1808267
Koellner-Clark, Karen; Borko, Hilda	2004	Establishing a Professional Learning Community among Middle School Mathematics Teachers	english	conference paper	http://emis.muni.cz/proceedings/PME28/RR/RR231_Clark.pdf
Leavitt, Della R.; Palius, Marjory F.; Babst, Robert D.; Donegan, Ryan; Lampkin, Jarrett L.; Smith, Michael; Whitford, Patricia A.	2013	Teachers Create a Professional Learning Community to be a Place of their Own.	english	journal article	https://doaj.org/article/cf46436b6aa74442a15947037fd35857
Leonard, Ann M.; Woodland, Rebecca H.	2022	Anti-Racism Is Not an Initiative: How Professional Learning Communities May Advance Equity and Social-Emotional Learning in Schools	english	journal article	http://dx.doi.org/10.1080/00405841.2022.2036058
Levy, Smadar; Bagno, Esther; Berger, Hana; Eylon, Bat-Sheva	2022	Professional Growth of Physics Teacher-Leaders in a Professional Learning Communities Program: The Context of Inquiry-Based Laboratories	english	journal article	http://dx.doi.org/10.1007/s10763-021-10217-7
Lücken, Markus	2012	Identifikation von Merkmalen erfolgreicher professioneller Lerngemeinschaften am Beispiel des Projekts "Biologie im Kontext" (bik)	german	contribution to a collection	https://www.fachportal-paedagogik.de/literatur/vollanzeige.html?FId=3162762
McLelland-Crawley, Rebecca	2014	Program Evaluation of a High School Science Professional Learning Community	english	monograph	https://scholarworks.waldenu.edu/tatations/1121/
Mohd Zabidi, Zuliana; Abdullah, Zuraidah; Sumintono, Bambang	2023	Exploring Teacher Collaboration: What's inside the Malaysian PLC Black Box?	english	journal article	http://dx.doi.org/10.1108/JPCC-03-2023-0020
Moulakdi, André; Bouchamma, Yamina	2020	Elementary Schools Working as Professional Learning Communities: Effects on Student Learning	english	journal article	https://files.eric.ed.gov/fulltext/EJ1254654.pdf
Oakley, Grace; King, Ronnel B.; Scarparolo, Gemma E.	2023	Digital Courseware Meets Professional Learning Community: Blended Learning to Improve the Teaching of Early Literacy in a Developing Country	english	journal article	https://doi.org/10.1080/13664530.2023.2175718
Rahman, S. M. Hafizur	2011	Influence of Professional Learning Community (PLC) on Secondary Science Teachers' Culture of Professional Practice: The Case of Bangladesh	english	journal article	https://eric.ed.gov/?id=EJ937637
Rahman, S. M. Hafizur	2012	Influence of Professional Learning Community (PLC) on Learning a Constructivist Teaching Approach (POE): A Case of Secondary Science Teachers in Bangladesh	english	journal article	https://eric.ed.gov/?id=EJ990369
Russell, John Lawson	2018	Professional Learning Communities and their Facilitation for Advancing Ambitious Teaching Practices.	english	monograph	https://doi.org/10.7916/D8SX7WND
Ryoo, Jean; Goode, Joanna; Margolis, Jane	2016	It Takes a Village: Supporting Inquiry- and Equity-Oriented Computer Science Pedagogy through a Professional Learning Community	english	journal article	http://dx.doi.org/10.1080/08993408.2015.1130952
Saito, Eisuke; Khong, Thi Diem Hang	2017	Not just for special occasions: supporting the professional learning of teachers through critical reflection with audio-visual information.	english	journal article	http://dx.doi.org/10.1080/14623943.2017.1361921
Shim, Soo-Yean; Thompson, Jessica	2022	Four Years of Collaboration in a Professional Learning Community: Shifting toward Supporting Students' Epistemic Practices	english	journal article	http://dx.doi.org/10.1002/sce.21704
Stammes, Hanna; Henze, Ineke; Barendsen, Erik; de Vries, Marc	2020	Bringing Design Practices to Chemistry Classrooms: Studying Teachers' Pedagogical Ideas in the Context of a Professional Learning Community	english	journal article	http://dx.doi.org/10.1080/09500693.2020.1717015
Stegmann, Karsten; Kastorff, Tamara; Poluektova, Ilona; Berger, Sonja; Kosiol, Timo; Reith, Sabrina; Förtsch, Christian; Rutkowski, Annemarie; Mohr, Matthias; Lindermayer, Christian; Aufleger, Monika; Traub, Dagmar; Haldenwang, Vera; Ufer, Stefan; Neuhaus, Birgit Jana; Bannert, Maria; Oechslein Karin, Lindner Martin; Nerdel, Claudia; Fischer, Frank; Gräsel, Cornelia	2022	Digitaler Wandel des Schulunterrichts durch professionelle Lerngemeinschaften.- Der Einsatz von Multiplikatoren zur Etablierung von Lerngemeinschaften	german	journal article	https://doi.org/10.21240/mpaed/49/2022.07.01.X
Szeto, Elson; Sin, Kenneth; Leung, George	2021	A Cross-School PLC: How Could Teacher Professional Development of Robot-Based Pedagogies for All Students Build a Social-Justice School?	english	journal article	http://dx.doi.org/10.1080/19415257.2020.1787201
Talkmitt, Marcia Joy	2013	K-12 Professional Learning Communities (PLCs) in a Rural School District on the High Plains of Texas: Mechanism for Teacher Support of Innovative Formative Assessment and Instruction with Technology (iFAIT)	english	monograph	https://eric.ed.gov/?id=ED555074
Thoma, Jennifer; Hutchison, Amy; Johnson, Debra; Johnson, Kurt; Stromer, Elizabeth	2017	Planning for Technology Integration in a Professional Learning Community	english	journal article	http://dx.doi.org/10.1002/trtr.1604

Appendix 2

Van Sickle, Meta; Perry, Lindsay; Capelloni, Alison	2017	University-Urban High School Partnership: Math and Science Professional Learning Communities	english	journal article	http://dx.doi.org/10.1111/ssm.12215
Vasinayanuwatana, Thanika; Teo, Tang Wee; Ketsing, Jeerawan	2021	Shura-Infused STEM Professional Learning Community in an Islamic School in Thailand	english	journal article	http://dx.doi.org/10.1007/s11422-020-09990-8
Vicheanpant, Thanyawich	2018	A Creation of Digital Professional Learning Community for Effectiveness Communication.	english	journal article	
Vossen, T. E.; Henze, I.; De Vries, M. J.; Van Driel, J. H.	2020	Finding the Connection between Research and Design: The Knowledge Development of STEM Teachers in a Professional Learning Community	english	journal article	http://dx.doi.org/10.1007/s10798-019-09507-7
Wong, Jocelyn L. N.	2010	What Makes a Professional Learning Community Possible? A Case Study of a Mathematics Department in a Junior Secondary School of China	english	journal article	http://dx.doi.org/10.1007/s12564-010-9080-6
Woolway, Jenny; Msimanga, Audrey; Lelliott, Anthony	2019	Continuous Collaborative Reflection Sessions in a Professional Learning Community: The Development of Grade 8 Natural Sciences Teachers' Reflective Practice	english	journal article	http://dx.doi.org/10.1080/18117295.2018.1555985

Complete citations of the literature used

Anderson, R. K., and Williams, M. (2023). Mathematics teachers' participatory patterns between face-to-face and virtual professional learning environments. *Journal of Mathematics Teacher Education, 26*(3), 295–320.

Baricaua Gutierez, S. (2016). Building a classroom-based professional learning community through lesson study: Insights from elementary school science teachers. *Professional Development in Education, 42*(2), 202–224. doi: 10.1080/19415257.2015.1119709

Barno, E., and Dietiker, L. (2022, November). *Collective curricular noticing within a mathematics professional learning community* [Conference presentation]. 44th Annual Meeting of the North American Chapter of the International Group for the Psychology of Mathematics Education, Nashville, TN, United States.

Berglund, H. (2022). Biology teachers' collaborative experiences: Benefits and difficulties in different contexts in relation to perceived value. *Journal of Science Teacher Education, 33*(8), 920–939. doi: 10.1007/s11422-022-10127-2

Bond, J. K. (2019). *Effects of professional learning communities on instructional revisions in secondary mathematics classrooms* [Doctoral dissertation, Walden University]. ProQuest Dissertations & Theses Global.

Bonsen, M., and Hübner, C. (2012). Unterrichtsentwicklung in professionellen Lerngemeinschaften [Instructional development in professional learning communities]. In T. Bohl and A. Nahrgang (Eds.), *Schul- und Unterrichtsentwicklung in der Schulpraxis* (pp. 99–112). Klinkhardt.

Brendefur, J. L., Whitney, B., Stewart, R. A., Pfiester, J., and Zarbinisky, J. (2014). Instructional learning teams: A case study. *Journal of Research in Education, 24*(1), 58–79.

Brig, L. (2014). How I learned the value of a true PLC. *Phi Delta Kappan, 96*(2), 78.

Brodie, K., and Chimhande, T. (2020). Teacher talk in professional learning communities. *African Journal of Research in Mathematics, Science and Technology Education, 8*(2), 118-130. doi: 10.46328/ijemst.v8i2.782

Casas, M. (2019). *Professional learning community (PLC) autonomy & trust: A cross case study.* [Doctoral dissertation, University of California, Irvine].

Chauraya, M., and Brodie, K. (2017). Learning in professional learning communities: Shifts in mathematics teachers' practices. *African Journal of Research in Mathematics, Science and Technology Education, 21*(3), 223–233. doi: 10.1080/0035919X.2017.1350531

Chauraya, M., and Brodie, K. (2018). Conversations in a professional learning community: An analysis of teacher learning opportunities in mathematics. *Pythagoras, 39*(1), a363. doi: 10.4102/Pythagoras.v39i1.363

Cheng, P. (2017). *Professional Learning Community (PLC): Technology Integration at a Title I Elementary School* [Doctoral dissertation, San Jose State University]. doi: 10.31979/etd.58wj-dk5q

Dalby, D. (2021). Professional learning through collaborative research in mathematics. *Professional Development in Education, 47*(4), 710–724. doi: 10.1080/19415257.2019.1665571

Appendix 2

Durr, T., Kampmann, J., Hales, P., and Browning, L. (2020). Lessons Learned from Online PLCs of Rural STEM Teachers. *The Rural Educator, 41*(1), 20-26. doi: 10.35608/ruraled.v4lil.555

Francis, D. S. (2011). *Improving a professional learning community at one elementary school: An action research study* [Doctoral dissertation, Florida Atlantic University]. FAU Digital Library.

Franks, D. (2012). *Mathematics professional learning communities: Opportunities and challenges in an elementary school context* [Doctoral dissertation, University of Delaware]. ProQuest Dissertations Publishing.

Hardman, E. L. (2012). Supporting professional development in special education with web-based professional learning communities: New possibilities with Web 2.0. *Journal of Special Education Technology, 27*(4), 17–31. doi: 10.1177/016264341202700402

Harris, E., and Rosenman, A. (2017). Discussing science in professional learning communities. *The Science Teacher, 84*(2), 47–52.

Higgs-Horwell, M., and Schwelik, J. (2007). Building a professional learning community: getting a large return on a small investment--I get by with a little help from my friends. *The English Journal, 96*(5), 113–116.

Hillman, P. C. (2018). *Vertically aligned professional learning communities as a keystone for elementary science teacher professional development, growth, and support* [Doctorial Dissertation, Columbia University]. Academic Commons. doi: 10.7916/D82N6JQH

Housen, M. (2018). *Disrupting high school students' mathematical disaffection through a professional learning community* [Doctoral dissertation, University of Pennsylvania]. ProQuest Dissertations and Theses Global.

Huggins, K. S., Scheurich, J. J., and Morgan, J. R. (2011). Professional learning communities as a leadership strategy to drive math success in an urban high school serving diverse, low-income students: a case study. *Journal of Education for Students Placed at Risk, 16*(3), 202–223.

Jones, M. B., II. (2018). *An exploration of teachers' lived experiences in professional learning communities in one Ohio urban school* [Doctoral dissertation, Franklin University]. ProQuest Dissertations & Theses Global.

Kasinathan, G., and Ranganathan, S. (2017). *Teacher professional learning communities: A collaborative OER adoption approach in Karnataka, India*. Zenodo. doi: 10.5281/zenodo.1094862

Kloser, M., Edelman, A., Floyd, C., Martínez, J. F., Stecher, B., Srinivasan, J. et al. (2021). Interrogating Practice or Show and Tell?: Using a Digital Portfolio to Anchor a Professional Learning Community of Science Teachers. *Journal of the Learning Sciences, 30*(2), 241–279. doi: 10.1080/1046560X.2020.1808267

Koellner-Clark, K., and Borko, H. (2004). Establishing a professional learning community among middle school mathematics teachers. In M. J. Høines and A. B. Fuglestad (Eds.), *Proceedings of the 28th Conference of the International Group for the Psychology of Mathematics Education* (Vol. 3, pp. 169–176). Bergen University College.

Leavitt, D. R., Palius, M. F., Babst, R. D., Donegan, R., Lampkin, J. L., Smith, M., and Whitford, P. A. (2013). Teachers create a professional learning community to be a place of their own. *Mid-Atlantic Education Review, 2*, 1–13.

Leonard, A. M., and Woodland, R. H. (2022). Anti-racism is not an initiative: How professional learning communities may advance equity and social-emotional learning in schools. *The Educational Forum, 86*(4), 362-377. doi: 10.1080/00405841.2022.2036058

Levy, S., Bagno, E., Berger, H., and Eylon, B.-S. (2022). Professional growth of physics teacher-leaders in a professional learning communities program: The context of inquiry-based laboratories. *Journal of Science Teacher Education, 33*(4), 438–458. doi: 10.1007/s10763-021-10217-7

Appendix 2

Lücken, M. (2012). Identifikation von Merkmalen erfolgreicher professioneller Lerngemeinschaften am Beispiel des Projekts „Biologie im Kontext“ (bik) [Identification of characteristics of successful professional learning communities using the example of the “Biology in Context” (bik) project]. In M. Kobarg (Eds.), *Lehrerprofessionalisierung wissenschaftlich begleiten. Strategien und Methoden* Prolog-Verlag, (pp. 99–112).

McLelland-Crawley, R. (2014). *Program evaluation of a high school science professional learning community* [Doctoral dissertation, University of Houston-Clear Lake]. ProQuest Dissertations & Theses Global.

Mohd Zabidi, Z., Abdullah, Z., and Sumintono, B. (2023). Exploring teacher collaboration: What's inside the Malaysian PLC black box? *Journal of Professional Capital and Community*. doi: 10.1108/JPCC-03-2023-0020

Moulakdi, A., and Bouchamma, Y. (2020). Elementary schools working as professional learning communities: Effects on student learning. *McGill Journal of Education, 55*(1), 166–187.

Oakley, G., King, R. B., and Scarparolo, G. E. (2023). Digital courseware meets professional learning community: Blended learning to improve the teaching of early literacy in a developing country. *Education and Information Technologies, 28*(12), 16405–16428.

Rahman, S. M. H. (2011). *Influence of professional learning community (PLC) on secondary science teachers' culture of professional practice: The case of Bangladesh*. UC Research Repository.

Rahman, S. M. H. (2012). *Influence of professional learning community (PLC) on learning a constructivist teaching approach (POE): A case of secondary science teachers in Bangladesh*. University of Canterbury.

Russell, J. L. (2018). *Professional learning communities and their facilitation for advancing ambitious teaching practices*[Doktorarbeit, Columbia University]. Academic Commons. doi: 10.7916/D8SX7WND

Ryoo, J., Goode, J., and Margolis, J. (2016). It takes a village: Supporting inquiry- and equity-oriented computer science pedagogy through a professional learning community. *Computer Science Education, 26*(1), 8–31. doi: 10.1080/08993408.2015.1130952

Saito, E., and Khong, T. D. H. (2017). Not just for special occasions: Supporting the professional learning of teachers through critical reflection with audio-visual information. *Reflective Practice, 18*(6), 795–807. doi: 10.1080/14623943.2017.1361921

Shim, S.-Y., and Thompson, J. (2022). Four years of collaboration in a professional learning community: Shifting toward supporting students’ epistemic practices. *Science Education, 106*(3), 674–705. doi: 10.1002/sce.21704

Stammes, H., Henze, I., Barendsen, E., and de Vries, M. (2020). Bringing design practices to chemistry classrooms: Studying teachers’ pedagogical ideas in the context of a professional learning community. *International Journal of Science Education, 42*(5), 778–797. doi: 10.1080/09500693.2020.1717015

Stegmann, K., Kastorff, T., Poluektova, I., Berger, S., Kosiol, T., Reith, S. et al. (2022). Digitaler Wandel des Schulunterrichts durch professionelle Lerngemeinschaften [Digital transformation of school lessons through professional learning communities]. *MedienPädagogik: Zeitschrift für Theorie und Praxis der Medienbildung, 49,* 1–25. doi: 10.21240/mpaed/49/2022.07.01.X

Szeto, E., Sin, K., and Leung, G. (2021). A cross-school PLC: How could teacher professional development of robot-based pedagogies for all students build a social-justice school? *Professional Development in Education, 47*(5), 844–860. doi: 10.1080/19415257.2020.1787201

Talkmitt, M. J. (2013). *K-12 professional learning communities (PLCs) in a rural school district on the high plains of Texas: Mechanism for teacher support of innovative formative assessment and instruction with technology (iFAIT)*[Doctoral dissertation, Texas Tech University]. Texas Tech University Libraries.

Thoma, J., Hutchison, A., Johnson, D., Johnson, K., and Stromer, E. (2017). Planning for technology integration in a professional learning community. *The Reading Teacher, 71*(2), 167–175. doi: 10.1002/trtr.1604

Appendix 2

Van Sickle, M., Perry, L., and Capelloni, A. (2017). University-urban high school partnership: Math and science professional learning communities. *School Science and Mathematics, 117*(3-4), 133–143. doi: 10.1111/ssm.12215

Vasinayanuwatana, T., Teo, T.W., and Ketsing, J.(2020). Shura-infused STEM professional learning community in an Islamic School in Thailand. *Cult Stud of Sci Educ 16*, 109–139 (2021). doi: 10.1007/s11422-020-09990-8

Vicheanpant, T. (2018). A creation of digital professional learning community for effectiveness communication. In *Proceedings of the 10th International Conference on Education Technology and Computers* (pp. 237–241). Association for Computing Machinery.

Vossen, T. E., Henze, I., De Vries, M. J., and Van Driel, J. H. (2020). Finding the connection between research and design: The knowledge development of STEM teachers in a professional learning community. *Journal of Science Teacher Education, 31*(1), 77–100.

Wong, J.L.N. (2010). What makes a professional learning community possible? A case study of a Mathematics department in a junior secondary school of China. *Asia Pacific Educ. Rev.* 11, 131–139. doi: 10.1007/s12564-010-9080-6

Woolway, J., Msimanga, A., and Lelliott, A. (2019). Continuous collaborative reflection sessions in a professional learning community: The development of Grade 8 natural sciences teachers' reflective practice. *Perspectives in Education, 37*(1), 108–123. doi: 10.1080/18117295.2018.1555985
